# Laparoscopic low anterior resection for hematogenous rectal metastasis from gastric adenocarcinoma: A Case Report

**DOI:** 10.1186/1477-7819-9-148

**Published:** 2011-11-11

**Authors:** Sang Woo Lim, Jung Wook Huh, Young Jin Kim, Hyeong Rok Kim

**Affiliations:** 1 Department of Colon and Rectal Surgery, Chonnam National University Hwasun Hospital, Gwangju, Korea

## Abstract

**Background:**

Gastric cancer is one of the most common malignancies in the world and is the second most common cause of cancer-related death in Korea. Colorectal metastases from gastric adenocarcinoma are known to be very rare. We report an unusual case of rectal metastasis of gastric adenocarcinoma.

**Case presentation:**

We report a case of a 43-year-old female patient with gastric cancer who first presented with epigastric pain. The endoscopic and radiologic findings were suggestive of Borrmann type III advanced gastric cancer with linitis plastica. Radical total gastrectomy with D2 lymph node dissection was performed. The pathology report was AJCC TNM Stage II gastric adenocarcinoma (T3N0M0). On follow up at 34 months after surgery, the patient complained of difficulty in defecation. On colonoscopy, a hard, indurated extraluminal mass was detected 7 cm proximal to the anal verge. The biopsy demonstrated chronic nonspecific colitis. Abdominal CT, rectal MRI and PET-CT revealed rectal metastasis from gastric cancer. Laparoscopic ultralow anterior resection with diverting ileostomy was performed. The pathology report was metastatic adenocarcinoma, and this diagnosis was identical to the gastric pathology reported in the previous pathology report. The patient was discharged after the 11^th ^postoperative day with no adverse events.

**Conclusion:**

Rectal metastasis from gastric cancer is known to be very rare. However, metastatic gastric adenocarcinoma should be considered as a differential diagnosis for patients presenting with a colorectal mass and a past history of gastric cancer.

## Background

Gastric cancer is one of the most common malignancies in the world and is the second most common cause of cancer-related death in Korea [1]. The known routes of distant metastasis after curative resection include: lymphatics (Virchow's node), peritoneal seeding (Krukenberg's tumor or Plummer's rectal shelf) or hematogenous spread (Schnitzler's metastasis) [2,3].

Colonic metastases from gastric adenocarcinoma are known to be very rare and such a hematogenous recurrence pattern of advanced gastric cancer with lymph node-negative and absence of lymphovascular invasion in the pathology after curative R0 resection has rarely been described.

We report an unusual case of rectal metastasis from gastric adenocarcinoma.

## Case Presentation

A 43-year-old woman who first presented with epigastric pain was diagnosed with gastric cancer. She had no family history of gastric cancer. Her past medical history revealed that she had undergone Cesarean section once. Laboratory findings showed mild anemia, with a hemoglobin level of 10.9 g/dL (reference range 12.3-16.5 g/dL). The endoscopic and radiologic findings revealed that the gastric mass was Borrmann type III advanced gastric cancer with linitis plastica.

She had undergone radical total gastrectomy with D2 lymph node dissection, and splenectomy in December 2007. The final pathology report after surgery demonstrated a diffuse, poorly differentiated adenocarcinoma. The surgical resection margins were clear. Thirty-five lymph nodes were harvested, and were found to be tumor-free, and spleen was not involved. Lymphovascular invasion was absent, although, perineural invasion was noted. The TNM staging according to the 6^th ^edition American Joint Committee on Cancer (AJCC) was stage II (T3N0M0)[4]. She was managed as an outpatient on a regular basis through physical examination, CBC and chemistry profile including vitamin B_12 _with monitoring every 3 months, abdominal CT every 6 months, and gastroduodenoscopy annually for 3 years. She had shown no evidence of disease recurrence in the 33 months prior to this episode. At thirty-four months after surgery, during a routine check-up, the patient complained of difficulty with defecation and constipation since the last.

**Figure 1:**
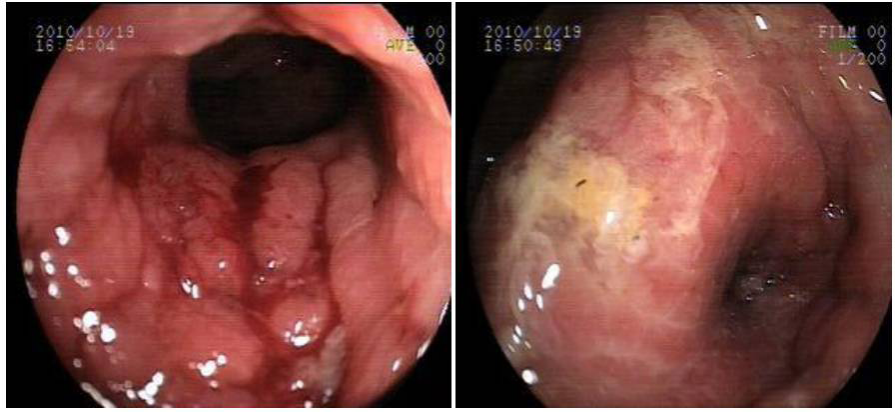
**Colonoscopic view of the circumferential rectal wall thickening located 7 cm above the dentate line and showing an edematous, erythematous, and nodular mucosa**.

1 month. On digital rectal examination and colonoscopy, hard induration and stenosis of the rectum, with an extraluminal mass 7 cm proximal to the anal verge, with edematous, erythematous, and nodular mucosa was detected (Figure 1). The colonoscopy-guided biopsy demonstrated chronic nonspecific erosive colitis.

On laboratory examination, serum carcinoembryonic antigen (CEA) level was 3.3 ng/mL (normal range: 5.0 ng/mL or less). The other serum tumor markers, including CA 19-9, were also within their normal ranges.

**Figure 2:**
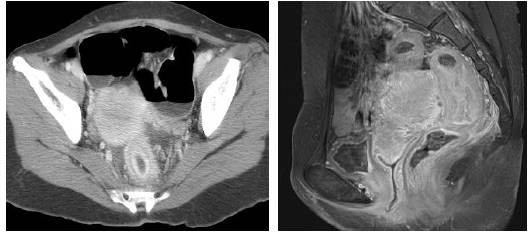
**A. Axial abdominal CT demonstrates concentric wall thickening, particularly in the inner enhancing layer with a target pattern**. B. MRI scan shows no evidence of tumor infiltration around the perirectal fat plane.

Abdominal computed tomography (CT) and rectal MRI revealed a 5 cm in length concentric wall thickening with enhancement in the mid-rectum about 6 cm proximal to the anal verge, and it had a target-like appearance (Figure 2). No evidence of tumor infiltration was observed around the perirectal fat plane. Overlying peritoneum showed thickening and enhancement suggestive of peritoneal seeding.

Her past medical history and radiologic findings suggested that metastasis to the rectum from gastric malignancy was more likely than primary rectal malignancy.

**Figure 3:**
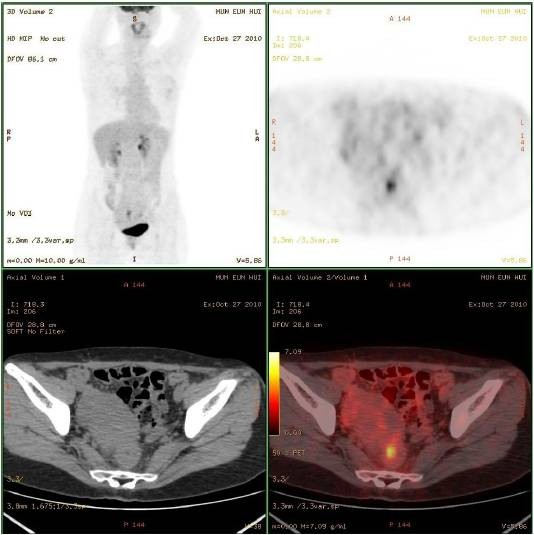
**FDG-PET showed hypermetabolism in the rectum**. The mean standardized uptake value calculated by FDG-PET for rectal tumor was 7.6.

On subsequent F-18-fluoro-deoxy-glucose (FDG) positron emission tomography (PET) scan, focal hypermetabolic lesion with the standardized uptake value (SUV) of 7.6 was detected in the upper rectum (Figure 3). No abnormal hypermetabolic activity was noted in the area of previous gastrectomy and anastomotic sites.

Finally, it was postulated that the rectal tumor was a metastatic tumor from gastric malignancy and peritoneal seeding in the pelvic cavity. Laparoscopic low anterior resection with diverting ileostomy was performed.

Briefly, the abdomen was entered using open (Hasson) method, and an 11 mm supraumbilical optic port was placed. A 12-mm port was placed in both the right and left lower quadrants and two 5-mm ports were placed about 10 cm proximal to each 12-mm port. The abdomen was insufflated with CO_2 _gas to a pressure of 12 mmHg. The moderate adhesions resulting from the previous gastrectomy were dissected and lysed with an ultrasonic dissector. After careful inspection of the liver and entire abdomen, a laparoscopic biopsy of the peritoneal mass was performed, and frozen sections were obtained for analysis. For operation of the rectum, a medial-to-lateral approach for laparoscopic colectomy was chosen and a high ligation of the inferior mesenteric artery and vein was performed, and the dissection was begun. Laparoscopic total mesorectal excision up to 2 cm distal to the rectal tumor was accomplished, and double stapling using endoscopic linear staplers and a circular stapler was performed for rectal transection and primary anastomosis. All procedures were in keeping with the oncologic principles.

**Figure 4:**
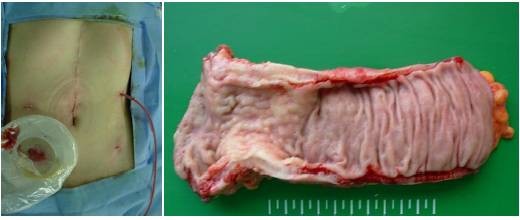
**Postoperative abdomen and surgical specimen of the rectal lesion**. The laparoscopy was performed via scar of the previous gastrectomy.

The OCTO port wound protector and retractor system (Dalim Medical, Daejun, Korea) and cylindrical vinyl film were used to cover the minilaparotomy for protection against cancer cell dissemination during specimen extraction. A diverting loop ileostomy was performed laparoscopically after reestablishing a pneumoperitoneum (Figure 4).

Intraoperative fresh frozen sections of paraaortic lymph nodes and peritoneal mass were obtained for analysis, and the findings were negative for malignancy.

The fresh specimen was about 17 cm in length on gross examination, and the rectal tumor size was about 4 × 4.5 cm^2 ^in size. The configuration of the lesion was of an ulceroinfiltrative mass. The safety resection margins were 10 cm proximally, 2 cm distally. Both the resected surgical margins were free from tumor cells. The circumferential margin after excision of the rectal tumor was 1.1 mm. The type of differentiation was of a poorly differentiated metastatic gastric cancer, invading all layers of the rectal wall up to pericolic soft tissue with focal mucosal invasion. Thirty-five lymph nodes were harvested, and all of them were found to be free from tumor cell invasion.

**Figure 5:**
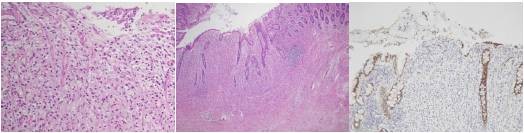
**Section of the rectal specimen showing metastatic gastric adenocarcinoma**. A, B. Hematoxylin and eosin stain C. CDX2 immunostain shows negativity.

The immunohistochemical staining for cytokeratin 7 (CK7), cytokeratin 20 (CK20), c-erb B2 and CDX2 were negative (Figure 5). The pathology report showed metastatic adenocarcinoma.

Oral feeding was started on the 3^rd ^postoperative day, and the patient was discharged after the 11^th ^postoperative day with no adverse events. After rectal resection, the patient was still being followed up for chemotherapy with the FOLFOX-4 regimen (Oxaliplatin 85 mg/m^2 ^IV on day 1, leucovorin 200 mg/m^2 ^IV on day 1, 5-FU 400 mg/m^2 ^IV bolus on day 1, and 5-FU 1,200 mg/m^2 ^continuous IV infusion on day 1 and 2) starting from December 2010. Her clinical course is being observed and she is exhibiting stable disease while the 12 cycles of the FOLFOX-4 regimen are being continued.

## Conclusion

The incidence of gastric cancer throughout the world is still high, and the incidence rate is increasing, especially in many Asian countries including Korea [1]. In clinical practice, lymph node metastasis, hematogenous spread, peritoneal seeding, and locoregional recurrence are the major routes for recurrence of gastric adenocarcinoma after curative resection [5,6].

Intestinal metastasis from gastric cancer is not common, although the most common cause of secondary neoplastic infiltration of the colon is gastric linitis plastica [7,8]. The main route of metastatic neoplastic infiltration involving the gastrointestinal tract is known to be hematogenous such that metastatic deposits invade the submucosal lymphatics and extend to form a linitis plastica appearance [9]. In the present study, although it is not clear whether this tumor was a genuine hematogenous metastasis to the rectum or it could be carcinomatosis associated with rectal invasion. Intraoperative fresh frozen sections of peritoneal mass were negative for malignancy. In addition, no grossly suspicious mass or nodules of carcinomatosis peritonei were found during laparoscopic exploration.

Also, the patterns of metastases in the intestinal and diffuse types of gastric cancer are known to be different, and the diffuse type of gastric cancer demonstrated a wider dissemination than the intestinal type. The intestinal type involved the liver more frequently [10]. Peritoneal metastasis, lymphatic permeation of the lung, and Krukenberg tumors were more common in the cases of diffuse type of gastric cancer. In the present study, the histology of primary gastric cancer was diffuse, poorly differentiated adenocarcinoma with linitis plastica that metastasized to the rectum three years later, and was comparable with atypical metastasis of gastric cancer.

The endoscopic features of luminal metastases are variable, but the typical characteristic features are diffuse circumferential wall thickening and stiffness which mimick those of linitis plastica such that the term secondary linitis plastica of the rectum has been used [11]. The overlying mucosa may appear to be normal, and will be negative for malignancy on mucosal biopsy, as was seen in the present study. A possible explanation for this may be that luminal metastases to the colorectum are usually known to preserve the mucosa, and the findings of colonoscopic biopsy may often be invalid [12,13].

CT appears to be the most feasible technique for detecting intestinal metastasis from gastric adenocarcinoma [12]. Intestinal metastasis from gastric cancer, especially of the linitis plastica type, most commonly shows target-like long segmental wall thickening with a characteristically thick inner enhancing layer on the helical CT. Jang et al reported that this type of metastasis also showed frequent association of peritoneal seeding and rare involvement of the liver, and emphasized that for CT of patients with gastric carcinoma or a past history of the disease, scrutiny of bowel for possible metastasis is needed.

The laparoscopic surgery has been an alternative to conventional open surgery in the treatment of colorectal carcinoma without compromising the oncologic outcome of laparoscopic colectomy [14,15]. In the present study, the surgery for rectal metastasis was performed laparoscopically. The issue of concomitant moderate degree of intra-abdominal adhesions after a previous history of radical gastrectomy and splenectomy has been overcome via advancement in laparoscopic surgical instruments and techniques used by experienced colorectal surgeons.

The clinical utility of CDX2 was confirmed to be fairly specific for the identification of adenocarcinoma of the gastrointestinal tract, particularly colorectal adenocarcinoma, in the primary and metastatic setting [16]. In 55% of gastric carcinomas, CDX2 expression was found by immunohistochemistry, with higher levels of CDX2 expressions noted in carcinomas with intestinal-type morphology [17]. In addition, the prognosis of gastric carcinoma patients with CDX2 positive expression is significantly better than that in patients with CDX2 negative expression [18]. In the present study, CDX2 negativity and cytokeratin 20 negativity suggested a non-colorectal origin of the tumor, rather than a colorectal malignancy.

After rectal resection, we used the FOLFOX regimen for systemic therapy due to our institutional preference. Other regimens such as DCF (docetaxel, cisplatin, and 5-FU), ECF (epirubicin, cisplatin, and 5-FU), irinotecan plus cisplatin, with other modifications could also be used.

In conclusion, we experienced a rare case of hematogenous rectal metastatic carcinoma from gastric adenocarcinoma which was treated successfully by laparoscopic surgery.

Colorectal metastasis from gastric cancer may occur, although it is very rare. When the symptoms of change in bowel habits such as abrupt hematochezia, or constipation are noticed, the possibility of metastases to the colon and rectum should be suspected, and the importance of endoscopic and CT surveillance for metastatic sites in the colon and rectum should be emphasized at postoperative check up during the follow-up period.

## Consent

Written informed consent was obtained from the patients for publication of this report and any accompanying images. A copy of the written consent is available for review with the Editor-in-Chief of this journal.

## Competing interests

The authors declare that they have no competing interests.

## Authors' contributions

SWL wrote the main manuscript and HRK performed the operation, revised the manuscript for important intellectual content, and gave the final approval for the version to be submitted for publication. All authors read and approve the final manuscript.
